# Population pharmacokinetic analysis of letermovir in adult hematopoietic cell transplant recipients

**DOI:** 10.1128/aac.00697-25

**Published:** 2025-08-22

**Authors:** Léna Royston, Carla Kunz, David Tonoli, Pierre Lescuyer, Dionysios Neofytos, Verena Gotta

**Affiliations:** 1 Division of Infectious Diseases, University Hospitals of Geneva 30574 , Geneva, Switzerland; 2 Division of Pediatric Pharmacology and Pharmacometrics, University of Basel Children's Hospital 30280, Basel, Switzerland; 3 Division of Laboratory Medicine, University Hospitals of Geneva, Geneva, Switzerland; Houston Methodist Hospital and Weill Cornell Medical College, Houston, Texas, USA

**Keywords:** cytomegalovirus, letermovir, therapeutic drug monitoring, hematopoietic cell transplantation, pharmacokinetic

## Abstract

We analyzed previously reported oral letermovir plasma concentration measurements from 40 allogeneic hematopoietic cell transplant (HCT) recipients using model-based pharmacometrics analysis. This analysis highlighted that the industry-sponsored phase III study model significantly over-predicted observed letermovir concentrations. Covariates associated with increased oral clearance (CL/F), possibly contributing to this observation, included: higher weight, younger age, and early use after HCT. The clinical significance and mechanistic explanation of relatively lower real-world exposure remains to be elucidated.

## INTRODUCTION

Letermovir is widely used as primary anti-cytomegalovirus (CMV) prophylaxis in CMV-seropositive allogeneic hematopoietic cell transplant recipients (HCTRs) (1). Despite its safety-efficacy balance, breakthrough clinically significant CMV (csCMV) infections and side effects occur (2). We recently conducted a prospective observational study assessing trough blood concentrations (C_trough_) of orally administered (PO) letermovir in allogeneic HCTRs, and its potential for therapeutic drug monitoring (3). Letermovir C_trough_ was associated with adverse events, while high intra- and interindividual exposure variability was observed. This post hoc pharmacometric analysis aimed to analyze pooled C_trough_ and non-C_trough_ samples of this study, to assess real-world pharmacokinetics compared to reported industry-sponsored data, and to explore covariates influencing variability in exposure (4).

This was a post hoc analysis of a prospective observational study, including adult allogeneic HCTRs receiving primary CMV prophylaxis with PO letermovir between 1 March 2020 and 20 April 2021 (3), administered once daily at 480 mg, or at 240 mg with cyclosporine. All patients signed an informed consent form, and the study was approved by the local Ethics Committee (ID 2020-00065).

We analyzed previously reported C_trough_ (24 ± 2 h post-dose, *n* = 217) and additional non-trough measurements (*n* = 79) from 40 patients, among six receiving cyclosporine, using model-based pharmacometric analysis. First, the industry-sponsored phase III model (4), forming the basis for the product label, was evaluated regarding its suitability (bias) to describe our real-world data. Second, the model was adjusted to fit our real-world data, comprizing PO letermovir only (vs PO and intravenous data in phase III model). Third, covariates potentially explaining variability in apparent oral clearance (CL/F, CL = systemic clearance, F = oral bioavailability) were explored in univariable analysis.

Initial phase III model simulations were done with Simulx version 2023R1, subsequent population pharmacokinetic modeling of real-world data with Monolix (Antony, France: Lixoft SAS, 2023). The modeled time scale was time after dose, and each patient visit was treated as an occasion at steady state. Data manipulation, general statistics, and figures were created using R (Version 4.3.3, R Development Core Team, Vienna, Austria, www.r-project.org).

**Fig 1:**
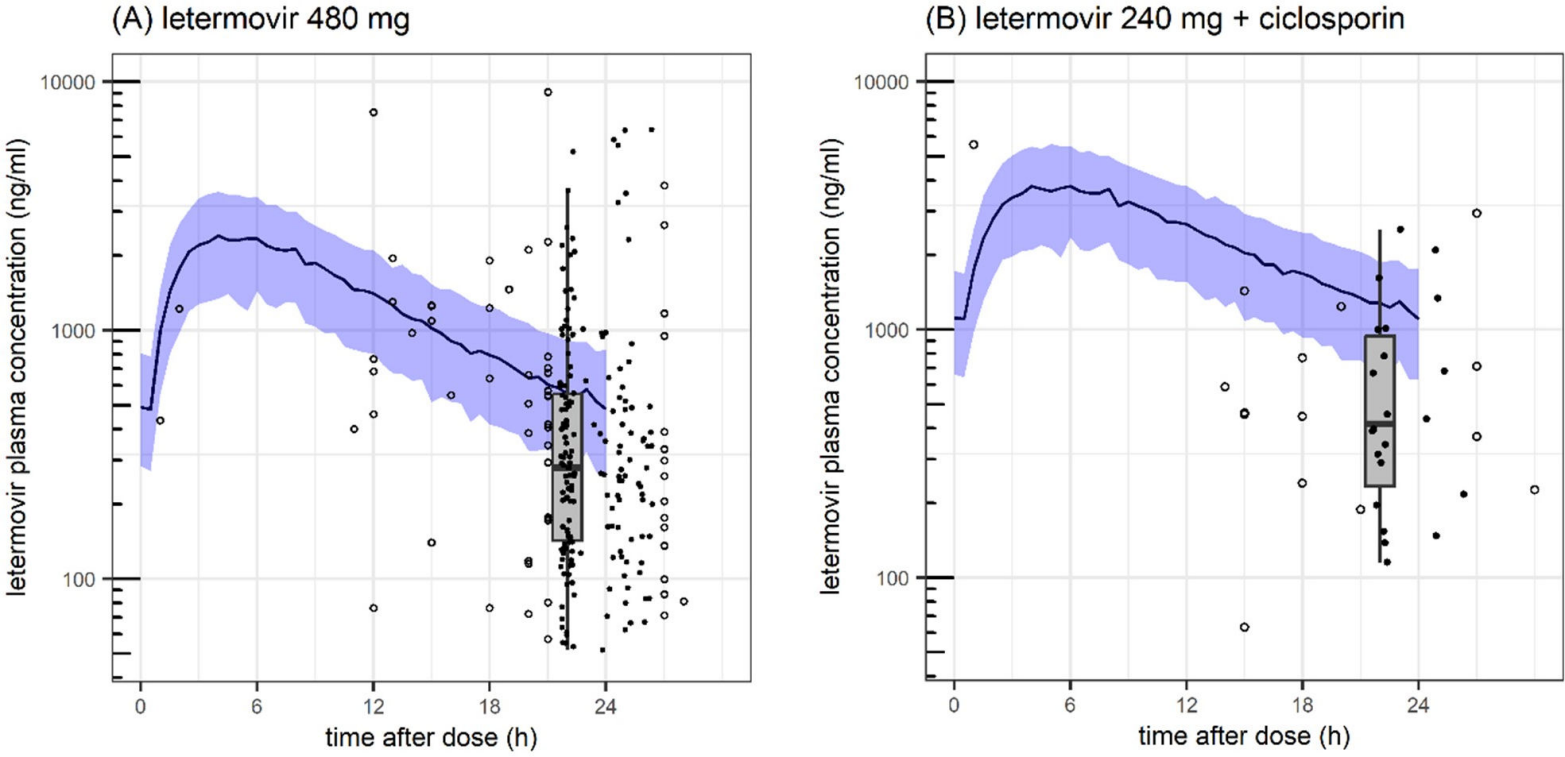
Adult oral letermovir pharmacokinetic data collected from clinical routine (*dots*: trough concentrations 22–26 h post-dose, *circles*: non-trough concentrations) contrasted with expected pharmacokinetic profile from industry-sponsored trials (4) (*solid line*: median, *blue shaded area*: interquartile range). *Boxplot*: summary of real-life trough concentrations (line: median, gray box: interquartile range). (**A**) letermovir 480 mg and (**B**) letermovir 240 mg with ciclosporin.

The phase III model significantly over-predicted observed real-world letermovir concentration measurements (Fig. 1). Median (IQR) predicted C_trough_ from the phase III model was 480 (263–838) ng/mL versus observed 260 (123–518) ng/mL for 480 mg/day (1.8-fold overprediction), and 1,104 (628–1,771) ng/mL versus 418 (235–945) ng/mL for 240 mg/day with cyclosporine (2.6-fold overprediction).

Refitting an adjusted model (simplified one-compartment with fixed absorption rate and lag time) significantly improved the model fit (AIC decreased from 4,830 to 3,739), with CL/F estimated to 26.5 L/h (relative standard error, RSE: 12.6%) without cyclosporin (reduced by 32% with cyclosporin). Inter-individual and inter-occasion variability in CL/F was high (standard deviation of log-transformed parameters estimated to 0.58 and 0.62, respectively) (Table 1). Goodness-of-fit plots are shown in the supplemental material.

**TABLE 1:** Comparison of population pharmacokinetic parameter estimates reported from industry-sponsored trials (“phase III model” (4) for non-asian subjects) and obtained estimates by fitting an adjusted model to our real-world data^*a*^^,^^*b*^

Parameter		Phase III model estimate from two-compartment model (RSE) [IIV/IOV]	Adjusted model estimate from one-compartmental model fitted to real-world data (RSE) [IIV/IOV]
Absorption rate constant	Ka (1 /h)	0.15 (18.9%) [0.72 (41%)/-]	0.15 (fixed) [0.72 (fixed)/-]
Absorption lag time	Tlag (h)	0.674 (6.8%)	0.674 (fixed)
Bioavailability			–
without CSA	F (-)	0.85 (12.0%)	
with CSA	F_CSA_ (-)	0.35 (10.5%)	
		[0.137 (24%)/0.197 (16%)]	
Central volume of distribution	V1 (L)	19.7 (5.8%)	–
Peripheral volume of distribution	V2 (L)	25.8 (15.6%) [0.23 (37%)/-]	–
Apparent oral volume of distribution	V/F	–	115.2 (12%) [0.23 (fixed)/-]
Inter-compartmental clearance	Q (L/h)	1.54 (14.4%)	–
Clearance			–
without CSA	CL (L/h)	4.8 (6.1%)	
with CSA	CL_CSA_ (L/h)	3.4 (9.7%)	
		[0.061 (17%)/-]	
Apparent oral clearance without CSA	CL/F	31.9 (calculated)	26.5 (12.6%) [0.58 (17%)/0.62 (7%)]
Decrease of apparent oral clearance with CSA	*f*_CSA_	−0.258 (calculated)	−0.38 (69.5%)
Residual error (%/μ/L)		52%/383 µg/L	29%/-

^*a*^ Values are given as population estimates (relative standard error, %). CSA: cyclosporin. IIV: inter-individual variability, IOV: inter-occasion variability, presented as standard deviation of log-transformed parameters.

^*b*^ '–’ indicates not applicable/not estimated.

The following covariates suggested association with increased CL/F in univariable analysis (*P* < 0.001 in likelihood ratio test, or regression coefficient estimated with good precision [RSE < 30%]): higher weight (allometric scaling), younger age, higher serum albumin, and vomiting. Decreased CL/F was associated with the presence of acute graft-versus-host disease (GvHD), prednisone, and posaconazole use. No associations were found with gender, glomerular filtration rate, liver enzymes (ALAT, ASAT, and bilirubin), C-reactive protein or documented infectious complications, absolute neutrophil or leukocyte count, nausea, mild to moderate diarrhea (<10× daily), pantoprazole treatment, or measured cyclosporin blood concentration (for those receiving ciclosporin).

Despite its increasing use as prophylaxis in transplant recipients, letermovir pharmacokinetic determinants remain poorly studied, with limited reports on real-world exposure.

This post hoc analysis, representing the first real-world pharmacometric investigation of letermovir to our knowledge, highlights discrepancies between reported industry-sponsored and real-world pharmacokinetics. Our cohort demonstrated significantly lower C_trough_, which was accounted for in an adjusted population pharmacokinetic model. Covariates potentially contributing to this observation through increased CL/F included patient characteristics (younger age and higher weight) and variables potentially associated with early use after HCT, such as absence of acute GvHD and steroid use, no antifungal prophylaxis with posaconazole, and low serum albumin. Some of these variables had already been associated in cross-sectional analysis (GvHD, prednisone, posaconazole) (3), while others were new in this model-based mixed-effect pharmacokinetic analysis (age, days since letermovir start, albumin, vomiting, and weight).

The reasons behind observed discrepancies remain unclear, as in- and exclusion criteria of industry-sponsored pharmacokinetic studies are not published in detail. Weight was a covariate in phase I, but not retained in phase III pharmacokinetics, possibly due to correlation with Asian ethnicity (4). Genetic variations in *SLCO1B1* (encoding for organic-anion transporter polypeptide 1B1 [OATP1B1] transporter) and *UGT1A1* can alter letermovir hepatic clearance, with the UGT1A1*6 variant being prevalent in Asian ethnicity (5). Younger patients may have generally better renal/hepatic function and clearance compared to older patients. GvHD triggers inflammation and cytokine release, including TNF-α and IL-1 (6). TNF-α can downregulate expression of OATP1B1 (7) and P-gp (8), potentially reducing letermovir clearance. In our cohort, acute GvHD increased from <20% during <5 weeks on letermovir to >30–40% between weeks 6 and 11, possibly resulting in a time-dependent decrease of letermovir oral clearance. Decreased oral bioavailability in the context of oral mucositis following conditioning (9) may result in increased CL/F. Albumin-mediated hepatic uptake of OATP substrates may explain a positive correlation between albumin and letermovir clearance (10).

Limitations of this analysis are associated with sparse sampling predominantly around trough and limited access to parenteral formulation during the study. This has reduced our ability to characterize drug absorption (rate and oral bioavailability) and distribution, and to dissociate factors reducing bioavailability from those increasing systemic clearance. Future work should explore transit models or variable absorption models, especially with richer sampling, and enhance confidence in the magnitude of decreased oral clearance with cyclosporin (estimated in this analysis to −38% with however large RSE). Simplifications to an oral one-compartment model appear justified given the lack of bi-phasic decline during 24-h dosing intervals (Fig. 1). High inter-individual and intra-occasion variability complicated robust covariate analysis, as convergence analysis indicated that identification of a “true” global minimum is a challenge. Hence, reported covariate associations should be viewed as hypothesis-generating rather than as confirmed predictors.

Despite these limitations, this analysis highlights relevant pharmacokinetic differences between the real-world setting and industry-sponsored trials, calling for further investigations. Although the association between letermovir concentrations and breakthrough csCMV infections remains unclear (3, 11), understanding pharmacokinetic determinants in the real-world setting could enable personalized letermovir dosing strategies to enhance efficacy and safety. The validity of our adjusted model should be confirmed in larger real-world studies, integrating oral and intravenous pharmacokinetic assessments.

## SUPPLEMENTAL MATERIAL

Fig. S1 aac.00697-25-s0001.docx Residual diagnostics from the applied phase III model (left) and fitted adjusted model (right).

ASM does not own the copyrights to Supplemental Material that may be linked to, or accessed through, an article. The authors have granted ASM a non-exclusive, world-wide license to publish the Supplemental Material files. Please contact the corresponding author directly for reuse.

## References

[1] Marty FM, Ljungman P, Chemaly RF, Maertens J, Dadwal SS, Duarte RF, Haider S, Ullmann AJ, Katayama Y, Brown J, Mullane KM, Boeckh M, Blumberg EA, Einsele H, Snydman DR, Kanda Y, DiNubile MJ, Teal VL, Wan H, Murata Y, Kartsonis NA, Leavitt RY, Badshah C. 2017. Letermovir prophylaxis for cytomegalovirus in hematopoietic-cell transplantation. N Engl J Med 377:2433–2444. doi:10.1056/NEJMoa1706640 29211658

[2] Royston L, Royston E, Masouridi-Levrat S, Vernaz N, Chalandon Y, Van Delden C, Neofytos D. 2021. Letermovir primary prophylaxis in high-risk hematopoietic cell transplant recipients: a matched cohort study. Vaccines 9:372. doi:10.3390/vaccines9040372 33921218 PMC8069238

[3] Royston L, Masouridi-Levrat S, Gotta V, Royston E, Pressacco-Brossier C, Abi Aad Y, Tonoli D, Karmime A, Jayo M, Van Delden C, Lescuyer P, Pfister M, Chalandon Y, Neofytos D. 2022. Therapeutic drug monitoring of orally administered letermovir prophylaxis in allogeneic hematopoietic stem cell transplant recipients. Antimicrob Agents Chemother 66:e0065722. doi:10.1128/aac.00657-22 35876579 PMC9380536

[4] Prohn M, Viberg A, Zhang D, Dykstra K, Davis C, Macha S, Sabato P, de Alwis D, Iwamoto M, Fancourt C, Cho CR. 2021. Population pharmacokinetics of letermovir following oral and intravenous administration in healthy participants and allogeneic hematopoietic cell transplantation recipients. CPT Pharmacometrics Syst Pharmacol 10:255–267. doi:10.1002/psp4.12593 33440077 PMC7965833

[5] Kobie J, Guo Z, Cho CR, Menzel K, McCrea JB, Blanchard R, Shaw PM. 2019. Pharmacogenetic analysis of OATP1B1, UGT1A1, and BCRP variants in relation to the pharmacokinetics of letermovir in previously conducted clinical studies. J Clin Pharmacol 59:1236–1243. doi:10.1002/jcph.1420 31022310

[6] Murray J, Stringer J, Hutt D. 2018 Graft-versus-host disease (GvHD), p 221–251. *In* Kenyon M, Babic A (ed), The European blood and marrow transplantation textbook for nurses: under the auspices of EBMT. Springer Copyright 2018, EBMT and the Author(s), Cham (CH). 31314221

[7] Fardel O, Le Vée M. 2009. Regulation of human hepatic drug transporter expression by pro-inflammatory cytokines. Expert Opin Drug Metab Toxicol 5:1469–1481. doi:10.1517/17425250903304056 19785515

[8] Fernandez C, Buyse M, German-Fattal M, Gimenez F. 2004. Influence of the pro-inflammatory cytokines on P-glycoprotein expression and functionality. J Pharm Pharm Sci 7:359–371. 15576018

[9] da Silva Ferreira AR, Märtson A-G, de Boer A, Wardill HR, Alffenaar JW, Harmsen HJM, Tissing WJE. 2021. Does chemotherapy-induced gastrointestinal mucositis affect the bioavailability and efficacy of anti-infective drugs? Biomedicines 9:1389. doi:10.3390/biomedicines9101389 34680506 PMC8533339

[10] Li N, Badrinarayanan A, Ishida K, Li X, Roberts J, Wang S, Hayashi M, Gupta A. 2020. Albumin-mediated uptake improves human clearance prediction for hepatic uptake transporter substrates aiding a mechanistic in vitro-in vivo extrapolation (IVIVE) strategy in discovery research. AAPS J 23:1. doi:10.1208/s12248-020-00528-y 33196949

[11] Prohn M, Cho CR, Viberg A, Dykstra K, Davis C, Sabato P, Stone J, Badshah C, Murata Y, Leavitt R, Fancourt C, Macha S. 2022. Exposure-response analyses of letermovir following oral and intravenous administration in allogeneic hematopoietic cell transplantation recipients. Clin Pharmacol Ther 111:485–495. doi:10.1002/cpt.2456 34674258

