# Supplementary material for: Population pharmacokinetic analysis of letermovir in adult hematopoietic cell transplant recipients
Source: Antimicrob Agents Chemother. 2025 Aug 22;69(10):e00697-25. doi: 10.1128/aac.00697-25 (PMC12486851; doi:10.1128/aac.00697-25)
Supplement: Fig. S1 — Residual diagnostics from the applied phase III model (left) and fitted adjusted model (right). [file aac.00697-25-s0001.docx]

**Supplemental material**

**
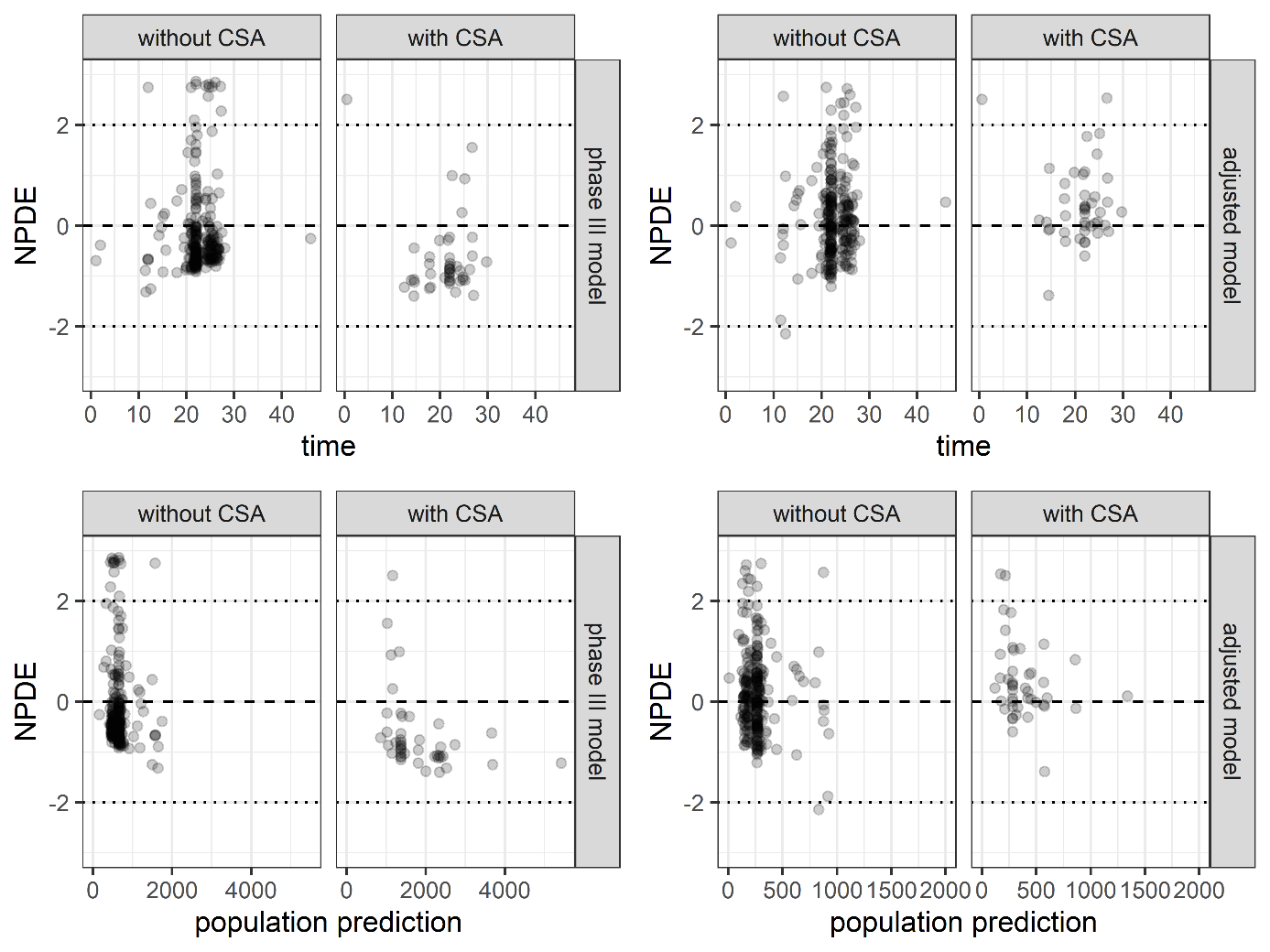
**

**Supplemental figure 1:** Residual diagnostics from the applied phase III model (*left*) and fitted adjusted model (*right*). NPDE: normalized prediction distribution errors.
